# Individual empowerment and community norm effects of engaging young husbands in reproductive health in rural India: findings from a pilot study

**DOI:** 10.21203/rs.3.rs-4376443/v1

**Published:** 2024-05-15

**Authors:** Nadia Diamond-Smith, Yogesh Vaishnav, Usha Choudhary, Payal Sharma, Ankur Kachhwaha, Tamera Panjalingam, Janelli Vallin, Debangana Das, Lakshmi Gopalakrishnan

**Affiliations:** University of California, San Francisco; Vikalp; Vikalp; Orange Tree Foundation; Orange Tree Foundation; University of California, Berkeley; University of California, San Francisco; Neerman; University of California, San Francisco

**Keywords:** Contraception, South Asia, male involvement, gender norms, couples relationships

## Abstract

**Background:**

Despite decades of a call to action to engage men in reproductive health, men are often left out of programs and interventions. In India, where half of pregnancies are reported as unintended, patriarchal gender norms and still dominant patterns of arranged marriages make engaging men in family planning and strengthening couples communication critical in increasing reproductive autonomy and helping young couples meet their reproductive goals. This study explores the feasibility and acceptability from the men’s perspective of the pilot of a gender transformative intervention for newly married couples in India.

**Methods:**

A pilot study was conducted of TARANG, a 4-month intervention for newly married women, with light touch engagement of husbands (4 sessions). A total of 41 husbands participated in the pilot, and we collected baseline knowledge and endline feasibility and acceptability data from them, along with in depth qualitative interviews with 13 men. The study was conducted in June-January, 2023.

**Results:**

Men had low levels of knowledge about biology, family planning, with the majority of men reporting that no one had provided them information about these topics. Most men wanted to delay the first birth by at least 2 years, yet less than a quarter had discussed childbearing plans with their partner or engaged in family planning methods. While all men reported high acceptability (satisfaction and usefulness), feasibility (participation) was low, with only 43% attending 2 or more sessions. Main barriers to participation included commitments due to work and migration. Men reported that the intervention led to improvements in their relationships with their wives, gave them a sense of empowerment, and led them to become resources for other men in their community.

**Conclusions:**

Men in these rural communities are not receiving the information that they need to meet their reproductive goals, however, they greatly desire this information and ways to improve relationships with their new wives. Such an intervention appears to have the potential to help change norms and spread information in the community and provide men with positive, life affirming feelings. Providing information through technology could address barriers to in-person engagement.

**Trial registration:**

clinicaltrials.gov (NCT06320964), 03/13/24

## Plain English summary

Despite many years of calls to engage men in reproductive health programming, there are still a paucity of interventions that engage men. In the Indian context, with high prevalence of arranged marriages, strict gender norms, and patrilocal marriage patterns where women move into their husbands family’s home, engaging men is especially important as they are critical decision-makers. With this in mind, we designed an intervention for newly married women, with an add on component for their husbands, to help young couples meet their reproductive goals. In this paper we report on the feasibility and acceptability of a 6 month pilot of this intervention with 40 newly married men in Rajasthan, India using mixed methods. We find that the intervention was highly feasibility and acceptable, and men felt that the intervention had value and should be considered. However, attendance was low due to work commitments and migration. The intervention appeared to change norms and increase knowledge about family planning and communication about childbearing. Men reported that the intervention led to improvements in their relationships with their wives, gave them a sense of empowerment, and led them to become resources for other men in their community. This suggests that engaging newly married men is possible, but interventions must be designed carefully to be sure to be able increase participation.

## Background

For decades, there has been a call to engage men in reproductive health across the globe.(1–3) Not only do men themselves use family planning, but they are often critical (or primary) decision-makers about whether their partners use family planning, especially within the marriage context. However, a recent systematic review exploring men’s engagement, and barriers to involvement, in reproductive health found only 47 papers published from 1994–2023.(4) Barriers to men’s engagement fell into four categories–those related to inclusive service provision, economic barriers, couple’s related factors, and socio-cultural norms around men’s involvement.

This call to men’s engagement in reproductive health is critical in India, where half of pregnancies are unintended and patriarchal gender norms favor men as decision-makers.(5) In India, as well as other countries, husbands dominate discussions about family planning and size in households and dictate their wives’ contraceptive use. This effect is magnified in rural areas and in family units where the wives are younger in age.(6) In a study analyzing male involvement in family planning in rural areas of India, investigators concluded that there are significant cultural barriers preventing husbands from engaging in dialogues with their wives regarding family planning and contraceptive use. In the context of Indian culture, family planning has been feminized in society, and engaging in family planning is seen as not masculine.(7) In another study in India, researchers conducted an analysis of male attitudes towards family planning and contraception, and their impact on women’s empowerment and engagement in family planning. The researchers observed that many husbands maintained the attitude that family planning and contraceptive use is unnecessary. Many husbands also did not agree with consensual sex practices, and believed that if their wives utilized contraceptives without their approval, it was appropriate to commit partner violence as a consequence.(8) All these attitudes among the male participants were measured to be associated with poor contraceptive use and family planning practices among women, highlighting the strong influence of husbands on family planning in households.(8)

Even though there has been an influx of programs globally and in India to promote family planning education and access to and affordability of contraceptives, the lack of engagement of husbands in family planning has been a barrier to the success of these programs. Given the significant role husbands play in family planning, comprehensive family planning programs should address the gender-based norms and couples communication to help women, men, and the couple-unit meet their reproductive goals.

Some interventions aiming to include men have been developed and tested in India, and elsewhere. For example, a randomized control study called CHARM was conducted, under which a family planning counseling intervention was delivered to husbands via single sessions and in combination sessions with both husbands and their wives. Contraceptive use in the intervention group increased from baseline to 9 months and 18 months more in the intervention than control group.(9) Among the study participants who had discussed contraception at home with their spouse, those who participated in CHARM were more likely to report continuing family planning discussions at both follow-up periods. The limitation of this study, however, was a lack of investigation into husbands’ support of their wives attending the program. Due to this limitation, the study was not able to adequately investigate the barriers to husbands support of family planning, and was not able to assess the barriers posed by husbands to family planning uptake.(9) Another study in India aimed to engage husbands in reproductive health, with a focus on increasing couple’s communication and addressing gender norms. While qualitative findings were promising, challenges arose with actually getting the men to participate in the intervention. (10) A similar intervention aiming to increase men that was implemented in India and Vietnam, also struggled with engaging men.(11)

Outside of India, a mixed-methods study in Kenya utilized a community-based family planning dialogue intervention to assess acceptability and use of family planning. In this study, researchers reported that women were more likely to utilize family planning if there was a greater level of spousal communication regarding this topic, and higher self-efficacy to discuss and use family planning in their households. (12) In Nepal, a mixed methods pilot study assessed the impact of a 4-month group intervention for newly married women, husbands, and mothers-in-law. From baseline to end of study, the percentage of mothers-in-law and married women that were opposed to utilizing family planning tools (i.e. contraceptives) to delay pregnancy decreased significantly (23% decrease among newly married women and 17% decrease among mothers-in-law).(13) This pattern was not observed among husbands, with their opposition to family planning methods increasing after being administered the intervention. This contradictory finding among the husbands indicates that further research needs to be conducted to uncover the limitations and barriers to family planning acceptance among husbands.

The goal of this paper is to describe the feasibility and acceptability of an intervention that aimed to engage newly married husbands into an intervention for newly married women in rural Rajasthan, India. Through a mixed methods design, we explore not only feasibility and acceptability, but men’s experiences with the intervention and how they felt that it impacted their lives and relationships.

## Methods

### The TARANG intervention:

TARANG (Transforming Actions for Reaching and Nurturing Gender Equity and Empowerment) meaning, ‘cascading waves’, in Hindi, was designed in partnership with a local NGO based in Udaipur, education/content development experts in Jodhpur, and the research study team through a series offormative phases (described elsewhere, under review)(14). The intervention is centered around newly married women, who received 16 sessions in a group format over a 6 month period. These sessions were aimed to improve reproductive health knowledge, promote agency and life skills, provide skills in enhancing couples communication, and address inequitable gender norms (described elsewhere, under review). Acknowledging that husbands are critical partners in women’s lives and reproductive health, we designed a light touch intervention with husbands, consisting of 4 group sessions on the following topics (Table 1):

We also included similar light touch sessions with mothers-in-law (4 group sessions).

Study site: The study was conducted across five villages in Kumbhalgarh block of Rajsamand district of Southern Rajasthan in India from July 2023 through January 2024. Kumbhalgarh block was chosen by the NGO partner based on their area of operations and cultural competency to work in these areas.

#### Sample

The sample size was based on feasibility given this was a pilot study. The eligibility criteria was focused around the newly married women, as the key intervention target. Thus, eligibility included: married women between the ages of 18 and 25 who had been living in their husband’s home at least for the past six months, cohabiting with mother-in-law, and had been married within the last one year. Eligibility for husbands included being over the age of 18. Individual private consent was sought from newly married women, and then the husbands. All participants who were approached agreed to participate but three participants did not meet eligibility criteria at baseline. A total of 45 potentially eligible households were approached and 42 newly married households were enrolled in the study at baseline. Three households could not be enrolled because two were migrants and one participant was a minor (below 18 years of age). All participants provided audio-recorded verbal informed consent before all rounds of data collection.

#### Data collection

##### Quantitative surveys

We collected quantitative data through close-ended surveys with participants before the launch of the intervention (baseline) and after the end of the intervention (endline). Sex matched trained enumerators conducted surveys (~60–70 minutes) using structured computer-assisted personal interviews (CAPI) on tablets.

##### Qualitative data

From the study participants enrolled in the study, we purposively selected 13 participants (Husbands) for in-depth interviews (IDIs) and 6 NGO staff implementing and managing the intervention. We attempted to interview participants who had less than 25% attendance as well as those with greater than 50% attendance to understand a diversity of perspectives and experiences. In October 2023, at the middle of the intervention period, in-depth interviews were led by two researchers (LG and YV) in participant’s preferred language (Hindi or Mewari), using an interview guide in a private setting at the participants’ homes or in their workplace site, depending on the convenience of the participants. We sought separate verbal consent from participants before conducting interviews. The average length of the interviews was ~30 minutes. Interviews were audio recorded and transcribed.

Throughout the period of the intervention, we also collected monitoring data through a mobile application form that was completed after every session by moderators. The form captured the attendance and reasons for missing the intervention sessions

##### Ethical approvals

Study protocols were reviewed and approved by institutional review boards at the University of California, San Francisco (IRB number: 22–37173), and the India-based Center for Media Studies (IRB00006230). This study was also registered on clinicaltrials.gov (NCT06320964).

#### Quantitative measures

We first describe respondent demographics (age, education, work status, caste, religion, type of marriage). We then describe baseline measures of men’s reports of if they discuss when to have children, how many children to have, gap between children, and family planning with their wives. We then describe baseline reproductive health knowledge measures around (1) when it is possible for a woman to get pregnant, (2) abortion legality, (3) recommended gap between children, and (4) types of family planning methods. Finally, we describe their stated timing for their first birth and if they are currently using family planning. All measures are from the baseline data.

Our primary feasibility measure included the proportion of participants who attended at least 50% of the intervention sessions. Acceptability data included satisfaction (proportion completely satisfied/somewhat satisfied with TARANG intervention) and usefulness (proportion who found TARANG intervention useful/somewhat useful). Other measures included willingness to recommend to a friend and a series of questions about how they thought that it impacted their life and relationships. Quantitative data were summarized as proportions using Stata 15.1. (15)

#### Qualitative themes and analysis

We analyzed in-depth interview transcripts line-by-line using a codebook. We developed a coding framework, deductively based on the interview guide, was iteratively refined with the addition of inductive codes following coding of initial transcripts. A team of three researchers initially double-coded at least 10% of the transcripts using the codebook in Dedoose version 9.0.107.(16) Themes were then developed using a thematic analysis.(17)

## Results

Husbands were, on average about 22 years old, ranging from 18–32 (Table 2). Over half had not completed high school, while 23.2% (N=5) had completed high school and 14.6% (N=6) had education beyond high school. About 20% were still pursuing further education. The majority (87.8%, N=36) were currently employed. Most identified as Hindu (97.6%, N=40), with a significant portion belonging to the scheduled tribe (63.3%, N=26). About a third reported having no prior acquaintance with their wives before marriage (N=15, 36.6%). Qualitative interviews were conducted with a subset of these same men, roughly representing the distribution of the full sample.

At baseline, few husbands reported having discussed certain family planning topics with their wives. Specifically, only 12.2% (N=5) had discussed the number of children to have, 19.5% (N=8) had discussed when to have the first child, 22% (N=9) had discussed the desired gap between children, and 17.1% (N=7) had discussed using family planning methods (refer to Table 3)

Knowledge regarding family planning was also low, with half (N=21, 51.2%) saying they didn’t know the recommended gap between children, and most (N=29, 70.7%) not knowing when a woman could get pregnant. Of those who provided an answer, 19.5% (N=8) said it was right after her period, and 7.3% (N=3) said it was during her period, with only 1 person mentioning between two periods. Most thought abortion was not legal (N=30, 73.2%), 22% (N=9) did not know, and only 2 knew that it was legal (4.9%).

Knowledge of temporary family planning methods was low. When asked to say if a list of 8 temporary methods were temporary or not, the mean score was 1.78, with no participant correctly listing more than 5 methods. Additionally, almost a third (N=12, 29.3%) did not list female sterilization as a permanent method, and almost two thirds (N=27, 65.9%) did not list male sterilization as a permanent method.

Regarding family planning intentions, only one respondent expressed a desire to have a baby immediately, and only 1 wanted a baby within less than a year. Among those who provided an answer, the majority (36.6%) expressed a preference to wait 24–36 months, followed by 16.7% preferring to wait 12–18 months, and 7.32% (N=3) preferring to wait over 3 years. However, despite these intentions, only 11.8% (N=4) of respondents were currently using family planning methods.

### 1. Increased knowledge

#### a. Increased knowledge about menstruation, birth spacing and contraceptives

Many of the husbands reported that they learned about the importance of birth spacing and using contraceptives through the TARANG sessions. They highlighted the challenges they could face if they did not have an appropriate gap between their children including not having time to focus on them, limited income, and potential health effects to their wives.

When the sir (moderator) talked about periods, I got to know. He talked about children, saying not to have them for two years. There should be a gap of four years between the first and second child because it would be challenging to manage children, not being able to focus on time, limited income, and a deficiency of blood in women. If you don’t want children, use condoms, and women have to take pills. Family members should come together and make decisions about what to do and what not to do. Bringing or doing anything improves relationships.

– husband 13, 24 years, 12^th^ standard, OBC

As mentioned in the quote above and below, learning about menstruation was one of the most valuable topics to the men in the sessions, perhaps because this is something that they had never had an opportunity to learn about before (as opposed to family planning which they might have had some exposure to through friends, mass media campaigns, or health workers).

The most beneficial one was about menstruation. It openly discussed things, and I found it good. It provided a lot of information

– Husband 7:25 years, BA, ST

The moderators also described how husbands shared that no one had ever talked to them about this type of information before.

Sometimes, the husbands share that no one has told them such a thing to date - it is very useful for them. They shared that there is no one else to whom they can ask all these things – not even neighbors. It seems that they need all these things. When I was doing the second and third sessions, there was [topic of] sex determination, fertility, during the session we talked to them and it came out that all these things were not known to them. Then it seemed that it was easy to see but they did not have such information.

– NGO staff

Not only did husbands report getting information for them, but the information led others in the community to seek their advice about family planning. One husband mentioned that he feels good that people come to him for information about family planning and that he can give them advice on the benefits of using it.

The benefit is that those who lack information seek advice from me; I provide them with information. I feel good that people ask me and take advice. They are understanding that using these methods should bring positive changes to their families. An example is that my friend has three children. And, when they were about to conceive their fourth child, I explained family planning methods to him. Initially, he thought it was useless, but later he agreed and started using these methods.

– Husband 7: 25 years, BA, ST

#### c. Increased knowledge about relationships, communication, and shared decision-making

Several husbands mentioned that one of the main benefits of the TARANG program was gaining knowledge about how to build/cultivate a healthy relationship with their wives. Husbands reiterated the importance of making decisions together, especially in relation to family planning and the selection of contraceptive methods.

There are many benefits, especially regarding relationships, family, and work. It helps in decision-making and knowing what to do. Both partners should be asked when choosing contraceptive methods and it should be used after taking advice from each other.

– 21-year-old husband with a BA education belonging to ST caste

Every topic is different, like, there should be consensus on anything in [our] family and we should be able to express our views to each other, I can express my views and my wife can also express her views. About anything. About children. In the topic of family planning, there should be a gap of three years between children, it is better than that, and it is also better in parenting. It is also good for [our] health. I like that I got the knowledge that I did not have earlier, and how scientifically this all happens.

– Husband 2: age 25 years, BA education, OBC

Expanding upon the value of communication, and how it helps with planning for the future, including around children, another husband explained:

There should be communication between husband and wife so that they can understand each other’s point of view.… There are many benefits [to receiving information as husband and wife]; it provides time to start planning for the future. Both should be on the same page about when to do it and when not to do it during this time.

– 24 year-old husband with a BA education belonging to OBC caste

#### c. Increased knowledge about consent and healthy spousal relationships

Related to building a healthy relationship, some husbands also mentioned that the TARANG program taught them to ask their wives for consent before engaging in intercourse. They also reported that if their wife is not feeling well, they should not insist on having sex.

We build relationships only with her consent. We don’t want to form relationships. If she is unwell, we shouldn’t insist on meeting. It’s good for our health and hers.

– 21 year old husband with a BA education belonging to ST caste

Men reported that these ideas around consent were new to them.

For the first time family planning was discussed, and there should be mutual consent, not coercion, on each other. I liked this. And also to understand each other’s feelings, do not impose anything. I did not hear of this concept before the session.

– Husband 2: 25 years, BA, OBC

One husband discussed how even if force or violence occurs, a couple can discuss that this was wrong and work to not let that happen again.

There should be an agreement between husband and wife; nothing should be done forcibly, and there should be no violence. If a mistake happens, it should be explained once not to do it again..

– Husband 9, 23, 12^th^ standard, SC

### 2. Impact

#### a. Increased communication between husbands and wives due to the sessions

Not only had husbands absorbed that communication, especially about family planning, was critical, but husbands reported that they talked to their wives about the information they received at the sessions and that they saw an improvement in communication. Before, either the husband or wife would feel shy to talk to one another, but now they could comfortably discuss when and how many children they want to have.

Besides information, there was also a significant health benefit. Knowing when to have a child, both information and benefits are there. Earlier, our family wasn’t as understanding, there was a bit of information, but now it’s fine. We discuss and share every minor and major thing by sitting together. In the evening, when going to bed, she asks where I went, and what work I did. I share the information that I had in the meeting. I got married 8–10 months ago, my wife came home three to four months ago. Either she would feel shy, or I would. Now, it’s not like that. Now we can comfortably sit and share what should be done, what should not be done, and is there a need for a child or not? Our age is not suitable now; it’s the age to earn money. Expenses need to be settled a bit; then we will see. For me, contraception is there and even if mistakes happen then Mala can be used by women; there are injections. We do take care of our bodies.

– Husband 6: age 23, BA, SC

Participants specifically mentioned how both of them (husband and their wives) receiving information was beneficial in promoting conversation and learning.

In my personal life, there has been a significant change in communication and behaviour with my partner. They have also attended sessions, and I have too, and we have learned a lot.

– husband 7: age 25 years, BA education, ST caste

Furthermore, there were instances where husbands sought their wives’ assistance in comprehending topics they did not fully grasp during the sessions. This mutual learning dynamic, with women imparting knowledge to men, is less common within the patriarchal gender norms prevalent in this setting.

I didn’t understand the topic of condoms, so I went home and asked my wife and she told me how to use them. We decided to use them together. We’ve talked about how many children to have two or three times. We didn’t talk about it earlier but after attending the meeting, we discussed this too. There has been a change understanding has come and we make decisions together.

– Husband 10, age 21, 10^th^ standard, OBC

The increased information for both husband and wife, and the fact that it led to more conversation and discussion together, was also noted to have enhanced their affection for each other..

Benefits, such as my wife learning about the changes in her body experiencing various aspects of living, and she is gaining information she didn’t have before. We openly discuss matters related to attending sessions as per the available time. It’s not forced. If there’s something to discuss, we talk willingly together. We have gained a lot of information from the sessions. There is a little difference in our way of living and a slight change in love. Earlier, there used to be a bit of irritation; their mindset wasn’t so open, and there was less communication. After attending sessions, she openly talks. The benefit in the family is that we use contraception, Mala-D etc. And the other benefit is that there are conversations; family members talk openly.

- Husband 7: age 25, BA, ST

Furthermore, some men reported that their new prospective and knowledge around consent had positive impacts on their sexual relationships.

There has been a lot of improvement regarding physical relations. What we did not know, how to live… how to behave with them, how to live with them, do only what both of us wish to do, do not do anything as per only my wish.

– Husband 5, 26 years, BA, SC

#### b. Male Empowerment

The combination of increased knowledge with the group format encouraging men to speak with others and in public seemed to have had larger impacts on some of the participants, providing men with a sense of empowerment. As one husband described:

By joining the group, the biggest benefit is that my power to speak increased, and my hesitation ended. I can share my thoughts in front of everyone. This has become my strength.

– Husband 6: age 23, BA education, SC

##### Feasibility and Acceptability

Overall, the mean number of sessions attended was 2.43 (out of 4) (Table 4). Only 9 husbands (22.50%) attended all 4 sessions, 25% (N=10) 3 sessions and 25% (N=10) 2 sessions. All men were completely (47.2%) or somewhat satisfied (52.8%) and most (N=24, 60%) found it very useful (with N=16, 40% somewhat useful). Most said they were very likely (N=15, 37.5%) or somewhat likely (N=23, 57.5%) to recommend it to a friend. Themes of empowerment that arose from the qualitative data also came out in the quantitative data, with 83% (N=30) saying there were more confident than before in making decisions about family planning. Over 80% reported improvements in decision-making and communication with their wives, and all felt they learned things they could apply in their daily life to some or a great extent.

While the overall reception was positive, it is clear that attendance, even to four sessions, was a challenge. Interviews highlighted the challenge to find a time that all men could come together in a group and participate. The main barrier was due to work responsibilities with some men working outside the village, further complicating participation. Additionally, disruptions due to festivals and household responsibilities also contributed to attendance issues..

The only problem is to manage time. Nothing else. There is some work pressure, as Diwali is also approaching, so people want their work to be done quickly. I was alone here. They took a session with me only.

– Husband 2, 25 years, BA, OBC

As is clear from the quote above, moderators adapted and sometimes provided the session to only 1 participant, or a very small group. This had impacts on the group dynamics, as explained by one of the NGO staff below, where it was harder to lead an engaging session with few people.

With a small number [of husbands], the moderator doesn’t get a sufficient environment. If two out of three husbands don’t respond, it feels like our session has ended. A session doesn’t become interesting with a small number, and there is a strong tendency to finish the session quickly.

– NGO staff

Husbands and moderators worked together to accommodate everyone’s work schedules, as is clear from the quote below, however, it remained a challenge to find a time to hold the groups, despite everyone’s willingness and effort.

We told sir {the moderator} that every guy who attends the session has work to do so, they go in the morning and come in the evening from work. So, we suggested sir to start the meeting at 8 PM, and even if it goes till 11 PM, no one will object. Everyone is free in the evening, and it’s convenient for us to attend.

– Husband 6: 23 years, BA, SC

## Discussion

Young newly married men in rural, tribal, Rajasthan are entering marriage with limited knowledge about reproductive health, but harbor significant interest and willingness to learn. These young men express a desire to delay having a baby, yet have little understanding about family planning methods and biological processes, often have incorrect information about methods and biology, and most were not using a contraceptive method. Studies across India for over 20 years have highlighted this lack of knowledge among men about fertility, family planning and reproduction, yet clearly this still remains a pressing issue (18). Additionally, the gap between men’s desire to delay/limit childbearing and their use of family planning has also been found in other studies in India.(19) Furthermore, they typically do not engage in discussions with their partners regarding the timing and desired number of children, nor do they discuss family planning methods. As was clear from the qualitative data, these men were eager for more information and felt like they had no one to talk to or ask questions of about these topics.

When provided with this relatively, light-touch intervention (comprising of 4 sessions to men, as a complement to a more intensive intervention for women) on reproductive health, family planning and couples communication, not only did their knowledge increase, but their self-reported behaviors changes, especially around increasing communication with their partners. They then observed other changes in their lives, like increased love and better sexual relations with their wives as a result. Intervening with newly married couples can help set couples on an alternate life course trajectory, not only allowing them to build and plan their family as they wish (which likely has implications for socio-economic stability and growth), but also to allow them to build a foundation early of strong communication, mutual respect, shared decision-making and more love and intimacy.

An unexpected finding is the impact of being part of a group session like this on men’s own sense of voice, agency, and empowerment. While we anticipated increased knowledge about family planning and couples relationships, which is part of empowerment (resources, using Kabeers framework), we did not anticipate that they would feel more able to speak up and have confidence.(20) Given the low educational, occupational, and social status (tribal, caste wise) of these men in their society, increasing their positive self-valuing could have positive impacts not only on them, but also their families and communities.

A related and somewhat surprising finding is that this empowerment was not only at the individual level, but had also spread to other community members. Some men felt that this intervention allowed them to become resources for others in the community, further spreading this knowledge and norms. Especially since the baseline found that men had such low levels of knowledge on these topics, the possibility that empowering one group of young men could lead to wider spread is promising and deserves further attention.

Despite high satisfaction, positive views on the impact on their lives, and sense of value in the intervention (high acceptability), feasibility (actually being able to bring together groups of men) remained a challenge through the intervention period. Overall participation was low, despite concerted efforts by moderators and participants to find times that worked and men’s eagerness to engage with the intervention. Other interventions in South Asia aiming to engage men in reproductive health have struggled with this same issue–men are busy working long hours and therefore its very hard to engage them, especially in a group.(10,11) Given this, we have restructured our intervention for the full randomized controlled trial to consist of one in person 2-hour workshop meeting followed by weekly WhatsApp videos on themes corresponding to women’s sessions. In these groups, the moderators will send videos, content, and lead discussions about topics that align with those in the women’s in-person sessions.

The mixed methods, longitudinal nature of this small pilot study is a strength, allowing us to measure baseline and endline knowledge, attitudes, and feasibility, and acceptability, as well as delver deeper into men’s experiences and barriers. However, this study has limitations. As a pilot study, the sample is small, and the study was conducted in only one district of Rajasthan, in only 4 villages. We were also not able to measure the impact of the intervention on knowledge, attitudes and behavior, but given the sample size of the pilot, this would have been descriptive at best. However, our larger cluster randomized controlled trial will examine the impact of the adapted TARANG intervention on men’s outcomes over an 18-month period (1 year post intervention).

## Conclusions

Despite decades of calls to engage men in reproductive health, men, especially young and socially disadvantaged men, are still lacking the information they desire. Engaging men presents a challenge, and it is understandable that many programs struggle to do so, but innovative and simple approaches may prove effective. Young husbands in this setting aspire to build healthier, more positive, and loving relationships with their wives, while also seeking greater understanding of fertility and contraception. Simple interventions such as this may go beyond educating men and improving their relationship, they may be able to positively empower men to be leaders of norm change in their communities.

## Figures and Tables

**Table 1:** Description of content of the four men’s sessions

Session 1	Name of the session	Details/highlights of the session
Session 1:	Love, Relationships and Expectations	Relationships run on love, not fear Consent is valid when It is decided voluntarily, enthusiastically and freely expressed It is given without any pressure (physical, mental or emotional) This is an informed consent It can be withdrawn at any time
Session 2:	Conception and Health	Highlights: Sex determination of fetus The man’s sperm determines the sex of the child in the womb, although it is not in the hands of both men and women. Pressure or violence in the family regarding the gender of the child is wrong
Session 3:	Contraception Methods	Highlights: Contraception cards, condoms There are different contraceptive methods according to different circumstances and different needs. Myths about condoms, vasectomy
Session 4:	How to choose family planning methods	Highlights: Family planning poster Family planning is the responsibility of husband and wife, it should be a personal decision.

**Table 2:** Demographics of the quantitative and qualitative samples

	Quantitative (N=41)	Qualitative(n=13)
Age (mean, range)	21.9 (18–32)	23.1 (19–25)
Education		
Less than 8^th^ grade	12 (29.27)	0 (0%)
8–11^th^ grade	18 (43.90)	3 (23.1%)
Completed high school (12^th^ grade)	5 (12.20)	2 (15.3%)
More than high school	6 (14.63)	8 (61.5%)
Not currently attending any more school	33 (80.49)	0 (0%)
Currently working	36 (87.80)	13 (100%)
Religion (Hindu, all others Islam)	40 (97.56)	Not available
Caste		
Schedule caste (SC)	6 (14.63)	5 (38.5%)
Schedule tribe (ST)	26 (63.41)	4 (30.8%)
Other backward caste (OBC)	5 (12.20)	4 (30.8%)
General category	3 (7.32)	0 (0.0%)
Did not know wife at all before marriage	15 (36.59)	Not available

**Table 3:** Baseline knowledge and practices around family planning discussions and use

Item	N (%)
**Communication about family planning**	
Ever discussed how many children to have	5 (12.20)
Ever discussed when to have first child	8 (19.51)
Ever discussed gap between children	9 (21.95)
Ever discussed which family planning method to use	7 (17.07)
**Knowledge**	
Don’t know recommended gap between children	21 (51.22)
Fertility window when a woman is most likely to get pregnant	
Don’t know	29 (70.73)
Between two periods	1 (2.44)
Right after her period has ended	8 (19.51)
During period	3 (7.32)
Is abortion legal in India	
No	30 (73.17)
Yes	2 (4.88)
Don’t know	9 (21.95)
Family planning methods	
Did not know female sterilization was permanent	12 (29.27)
Did not know male sterilization was permanent	27 (65.85)
Temporary FP score (total possible 8), mean, range	1.78 (0–5)
**Desires and Behaviors**	
Desired time between marriage and the first baby	
Immediately	1 (2.44)
<1 year	1 2.44
12–18 months	6 (16.67)
24–36 months	15 (36.58)
3+ years	3 (7.32)
Don’t know	15 (36.59)
Currently using any family planning method	4 (11.76)

**Table 4:** TARANG intervention perspectives from husbands who participated in the intervention.

	N (%) (or mean)
Mean number of sessions attended (range 1–4)	2.425
Proportion of participants who attended at least 50% of the sessions (primary feasibility outcome)	18 (43.9%)
How satisfied were you? (acceptability outcome)	
Completely	17 (47.22)
Somewhat	19 (52.78)
How useful were the discussions and topics ? (acceptability outcome)	
Somewhat useful	16 (40.00)
Very useful	24 (60.00)
How likely it is that you will recommend a friend to join?	
Very unlikely	1 (2.50)
Somewhat likely	23 (57.50)
Very likely	15 (37.50)
Can’t say	1 (2.50)
How do you feel about having discussion on having children and your health after TARANG?	
More confident than before	30 (83.33)
Just as confident as before	6 (16.67)
Did you learn info from TARANG that could be applied in your daily life?	
Can apply to some extent.	24 (60.00)
Can apply to a great extent.	16 (40.00)
Have you observed changes in decisions about having children after TARANG	
Some positive changes	32 (88.89)
More positive changes	3 (8.33)
Can’t say	1 (2.78)
Impact of TARANG on communication with your wife about family planning?	
A little negative impact	2 (5.00)
A little positive impact	32 (80.00)
A very positive impact	6 (15.00)

## Data Availability

Data will be put on a public repository as soon as results from the main analysis are published
